# Association analysis of miRNA-related genetic polymorphisms in miR-143/145 and *KRAS* with colorectal cancer susceptibility and survival

**DOI:** 10.1042/BSR20204136

**Published:** 2021-04-22

**Authors:** Danyang Wang, Qingmin Liu, Yanjun Ren, Yan Zhang, Xin Wang, Bing Liu

**Affiliations:** 1 Department of Colorectal Surgery, The First Affiliated Hospital, College of Medicine, Zhejiang University, Hangzhou, Zhejiang, China; 2 Department of Chronic and Non-communicable Disease Prevention and Control, Hangzhou Center for Disease Control and Prevention, Hangzhou, Zhejiang, China; 3 Department of Pharmacy, Ziyang Community Health Service Center, Hangzhou, Zhejiang, China

**Keywords:** colorectal cancer, kras, mir-143/145, single nucleotide polymorphisms, survival, susceptibility

## Abstract

Background: There is accumulating evidence of aberrant expression of miR-143 and miR-145 and their target gene *KRAS* in colorectal cancer (CRC). We hypothesize that single nucleotide polymorphisms (SNPs) within or near mRNA–microRNA (miRNA) binding sites may affect miRNA/target gene interaction, resulting in differential mRNA/protein expression and promoting the development and progression of CRC. Methods: We conducted a case–control study of 507 patients with CRC recruited from a tertiary hospital and 497 population-based controls to assess the association of genetic polymorphisms in miR-143/145 and the *KRAS* 3′ untranslated region (3′UTR) with susceptibility to CRC and patients’ survival. In addition, genetic variations of genomic regions located from 500 bp upstream to 500 bp downstream of the miR-143/miR-145 gene and the 3′UTR of *KRAS* were selected for analysis using the Haploview and HaploReg software. Results: Using publicly available expression profiling data, we found that miR-143/145 and *KRAS* expression were all reduced in rectal cancer tissue compared with adjacent non-neoplastic large intestinal mucosa. The rs74693964 C/T variant located 65 bp downstream of miR-145 genomic regions was observed to be associated with susceptibility to CRC (adjusted odds ratio (OR): 2.414, 95% CI: 1.385–4.206). Cumulative effects of miR-143 and miR-145 on CRC risk were observed (*P*_trend_=0.03). Patients having CRC carrying variant genotype TT of *KRAS* rs712 had poorer survival (log-rank *P*=0.044, adjusted hazard ratio (HR): 4.328, 95% CI: 1.236–15.147). Conclusions: Our results indicate that miRNA-related polymorphisms in miR-143/145 and *KRAS* are likely to be deleterious and represent potential biomarkers for susceptibility to CRC and patients’ survival.

## Introduction

Colorectal cancer (CRC) is one of the most commonly occurring malignancies worldwide. According to The Global Burden of Cancer 2013, colon and rectal cancer ranked third for cancer incidence and fourth for cancer deaths [1]. In China, incidence and mortality statistics of CRC for 2014, published by the National Cancer Center, showed a similar trend, with CRC ranking the third and fifth place for cancer incidence and cancer deaths, respectively [2].

The development of CRC is a multifactorial and multistep process involving the gain and maintenance of specific genomic alterations [3]. Over the past few decades, many associations have been identified between the variation of protein-coding genes and CRC. In the recent years, high-resolution maps of the human transcriptome have led to the discovery of a large number of non-protein-coding RNA genes and brought about a paradigm shift in our understanding of the function of variations in non-coding RNAs (ncRNAs) [4]. The ncRNAs include a class of short RNA molecules termed microRNAs (miRNAs), which are endogenous small ncRNAs that repress protein-coding genes by binding to target sites in the 3′ untranslated region (3′UTR) of mRNAs. These miRNAs are involved in the regulation of almost all physiological and pathological processes, including cell proliferation, differentiation, and apoptosis [5].

MiR-143 and miR-145, which are located close to each other on 5q33, are co-transcribed from a single promoter and generate a primary transcript containing both miRNAs [6]. In 2003, miR-143 and miR-145 were reported to be down-regulated in colorectal tissue for the first time [7]. Subsequently, a series of studies confirmed these results [8–10]. Decreased expression of these two miRNAs is involved in various cancer-related events, including proliferation, invasion, and migration, suggesting that they have anti-tumorigenic activity [11–13]. The *KRAS* oncogene is an important upstream mediator of the MAPK pathway, and its overexpression can lead to increased activation of the RAF/MEK/MAPK pathway, thereby promoting tumorigenesis [14]. *KRAS* is an important target of miR-143/145, which has been identified not only by computational predictions using software such as TargetScan, miRanda, and PicTar, but also by experimental validation [15,16].

Mutations in either miRNAs or their co-expressed miRNA binding sites are often deleterious, which can affect miRNA/target gene interaction, resulting in differential mRNA or protein expression and increased susceptibility to common diseases [17]. This view was supported by some studies of miRNA-related genetic alterations with different types of cancer, including CRC [18–20]. However, published evidence for genetic variations of miR-143/145 and the 3′UTR of *KRAS* with CRC susceptibility are limited and not comprehensively investigated. Therefore, we conducted a case–control study to assess the association between these candidate biomarkers with risk of having CRC.

## Materials and methods

### Study population

The present study was conducted in Hangzhou City, Zhejiang Province, China. Five hundred and seven patients with CRC and four hundred and ninety-seven cancer-free controls were enrolled in the study from May 2014 to May 2015. These patients were recruited from a tertiary hospital in Hangzhou, Zhejiang Province, China. Eligible cases were newly diagnosed and histologically confirmed CRC without pre-operative radiotherapy or chemotherapy. The control population was recruited from among the individuals who came to the community health service centre for medical examinations. The controls had no cancer history or intestinal diseases. All participants were Han Chinese and had lived in Zhejiang Province for more than 20 years.

The study was approved by the Medical Ethical Committee of Hangzhou Center for Disease Control and Prevention (No. 2019-4). All participants had signed informed written consent. Face-to-face interviews were conducted by trained interviewers who administered a structured questionnaire asking about demographic characteristics, family history of cancer, previous medical history, and lifestyle-related factors. Smoking history was defined as having smoked at least one cigarette per day for more than 1 year. Chronic alcohol drinking or tea drinking was defined as having consumed an alcoholic drink or tea for at least once per day for more than 3 months.

### Polymorphism selection and genotyping

First, single nucleotide polymorphisms (SNPs) of genomic regions located from 500 bp upstream to 500 bp downstream of the miR-143/miR-145 gene and the 3′UTR of *KRAS* were downloaded from 1000 Genomes (http://www.internationalgenome.org/) if they had minor allele frequency > 0.05 within the Southern Han Chinese (CHS) population. Then, tag single nucleotide polymorphisms (tagSNPs) representing SNPs with the pairwise correlation of r^2^ > 0.8 were further selected using the tagger algorithm implemented in the Haploview software. The function of the tagSNPs was predicted using RegulomeDB and HaploReg. Finally five polymorphisms were selected for study: *KRAS* rs712, rs1137196, miR-143 rs41291957, miR-145 rs74693964, and rs80026971. Detailed information regarding the selected SNPs is listed in Supplementary Table S1.

In all the patients, 5 ml of peripheral blood was collected in anticoagulation tube at the time of pre-surgery examination and stored in −80°C refrigerator. Genomic DNA was extracted from peripheral blood samples using a magnetic bead method with KingFisher Flex (Thermo Scientific, U.S.A.). The concentration and purity of the DNA samples were determined using a NanoDrop2000 spectrophotometer (Thermo Scientific, U.S.A.). Genotyping was performed using the Agena MassArray Genotyping Platform (Agena Inc. San Diego, CA, U.S.A.). Five percent blinded samples were repetitively genotyped and a negative control was interspersed throughout the genotyping assays. The detection rates of all SNP genotyping assays were ≥96%. The concordance rates for duplicated samples were 100%.

### Gene expression analysis

The miR-143/145 microarrays were downloaded from the Gene Expression Omnibus (GEO) database (www.ncbi.nlm.nih.gov/geo), accession no. GSE38389. From this dataset, 66 paired samples from rectal tumor tissue and non-neoplastic large intestinal mucosa samples were collected, and miRNA expression profiles were detected using the GPL11039 platform (Exiqon miRCURY LNA microRNA array v.9.2 Extended Version).

We used Oncomine (www.oncomine.org, last accessed on 15 March 2021) to conduct a meta-analysis for *KRAS* gene expression. We extracted the qualified datasets by key words as follows: Gene ‘*KRAS*’; Cancer type: ‘Colorectal cancer’; Analysis type: ‘Cancer vs. Normal Analysis’; Data Type: ‘mRNA’ from the the Oncomine database. There are seven arrays (Ki colon, Kaiser Colon, Skrzypczak Colorectal 2, TCGA Colorectal, Skrzypczak Colorectal, Hong Colorectal, Gaedcke Colorectal) including 578 CRC cases and 179 controls involved in the meta-analysis. Furthermore, We used the UALCAN database (http://ualcan.path.uab.edu/) to analyze the *KRAS* mRNA expression based on the TCGA CRC dataset.

### CRC death surveillance

A follow-up survey of the patients with CRC was performed with Hangzhou household registration using the Cancer Registration System and Death Surveillance System of Hangzhou Center for Disease Control and Prevention. The date of censorship was 1 January 2019. We applied the identification card (ID) number and name of patients with CRC to match in order to acquire the survival outcome from the Surveillance Systems. The date and cause of death were recorded for the survival analysis.

### Statistical analysis

A two-sided Student’s *t* test was used to compare the differences in the quantitative data, and a chi-square test was used to compare categorical data between the two groups. Departures from Hardy–Weinberg equilibrium were tested using goodness-of-fit chi-square test. Multivariate logistic regression analysis was performed to explore the association between the selected SNPs and risk of CRC with adjustment for age, gender, and family history of cancer. A likelihood ratio test was used to assess the interaction effects between the SNPs and smoking with respect to CRC. The Cochran–Armitage test was used for trend analysis. Kaplan–Meier survival analysis and log rank test were used to assess survival outcome, that is, overall survival (OS) of the patients in relation to the genotypes. Multivariate Cox regression analysis was performed to calculate relative risk [hazard ratio (HR)] and 95% confidence interval (CI) associated with genetic polymorphisms from cancer diagnosis until the end of the study or death. Statistical analyses were performed using the Statistical Package for the Social Sciences (SPSS) version 25 (IBM, New York, U.S.A.). A *P*-value <0.05 was considered statistically significant.

## Results

### Characteristics of the study population

Of the 507 patients with CRC patients, 209 had colon cancer cases and 298 had rectal cancer cases. The baseline characteristics and lifestyle factors are shown in Table 1. There was no significant difference in age between the cases patients and the controls, but the proportion of males was higher in the cancer group (64.89 vs. 57.95%). In addition, patients with CRC were more likely to have a lower education level and lower body mass index (*P*<0.05) than the controls. CRC patients also reported higher percentages of family history of cancer and history of appendicitis in comparison with controls (*P*=0.034, *P*<0.001, respectively). On the other hand, no significant differences were found between the patients with CRC and control groups with respect to tobacco smoking, alcohol drinking, or tea drinking.

**Table 1:** Baseline characteristics of study population

Characteristics	Cases (*n*=507)	Controls (*n*=497)	Statistics	*P-value*
Age (years), (mean ± SD)	62.55 ± 11.88	62.75 ± 11.99	0.264	0.792
Gender, *n* (%)				
Male	329 (64.89%)	288 (57.95%)	5.109	0.024
Female	178 (35.11%)	209 (42.05%)		
Former BMI (kg/m^2^), *n* (%)^†^				
<18.5	18 (3.56%)	15 (3.27%)	13.038	0.001
18.5–23.9	298 (58.89%)	219 (47.71%)		
≥23.9	190 (37.55%)	225 (49.02%)		
Education level, *n* (%)				
Illiterate	63 (12.45%)	25 (5.20%)	63.428	<0.0001
Primary school	182 (35.97%)	91 (18.92%)		
Middle school and above	261 (51.58%)	365 (75.88%)		
Marital status, *n* (%)				
Married	504 (99.41%)	412 (84.08%)	78.418	<0.0001
Unmarried	3 (0.59%)	78 (15.92%)		
Family history of cancer, *n* (%)				
No	408 (80.47%)	425 (85.51%)	4.511	0.034
Yes	99 (19.53%)	72 (14.49%)		
History of appendicitis				
No	477 (94.08%)	489 (98.39%)	12.787	<0.0001
Yes	30 (5.92%)	8 (1.61%)		
Smoking, *n* (%)^*^				
No	324 (63.91%)	338 (69.12%)	0.353	0.552
Yes	183 (36.09%)	151 (30.88%)		
Alcohol consumption, *n* (%)^*^				
No	359 (70.81%)	353 (71.03%)	0.772	0.380
Yes	148 (29.19%)	144 (28.97%)		
Tea consumption, *n* (%)^*^				
No	230 (45.36%)	239 (48.09%)	0.046	0.830
Yes	277 (54.64%)	258 (51.91%)		

* Cochran–Mantel–Haenszel test, adjusted by sex.

† BMI of 5 years before investigation.

### mRNA expression analysis of miR-143, miR-145, and *KRAS*

**Figure 1:**
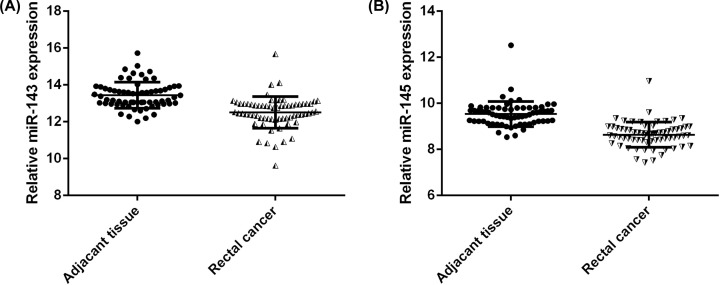
MiR-143, miR-145 expression in samples for rectal cancer and adjacent normal mucosa (**A**) MiR-143 expression in rectal cancer and matched normal mucosa in the rectal cancer study identified from the GEO profile database (accession number GSE38389). Of the 66 tumor biopsies and corresponding matched mucosa samples, 50 were under-expressed (log2-fold difference < −0.5) and four highly expressed (log2-fold difference > 0.5) in rectal cancer tissue compared with adjacent normal controls (*P*-value for paired *t* test <0.001). (**B**) MiR-145 expression in rectal cancer and matched normal mucosa in the rectal cancer study identified from the GEO profile database (accession number GSE38389). Of the 66 tumor biopsies and corresponding matched mucosa sample, 50 were under-expressed (log2-fold difference < −0.5) and one highly expressed (log2-fold difference > 0.5) in rectal cancer tissue compared with adjacent normal controls (*P*-value for paired *t* test <0.001).

We extracted published microarray data from GEO datasets GSE38389 and compared the mRNA expression of miR-143, miR-145 between rectal cancer tissue and adjacent non-neoplastic large intestinal mucosa. As shown in Figure 1, miR-143 expression was under-expressed (log2-fold difference < −1) in 26 out of 66 matched pairs of rectal tumor samples and non-neoplastic samples (*P*-value for paired *t* test <0.001). MiR-145 showed the same trend, with decreased expression (log2-fold difference < −1) in 28 out of 66 pairs of samples (*P*-value for paired *t* test <0.001) (Figure 1A,B).

**Figure 2:**
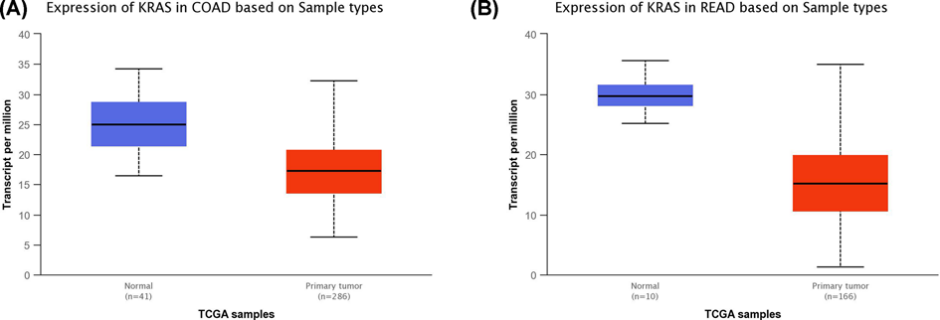
*KRAS* mRNA expression in CRC from TCGA dataset (**A**) *KRAS* mRNA expression in colon cancer tissue and adjacent normal mucosa. Box plot and *P*-value were produced using UALCAN. *P*=9.41025035672283E-13. (**B**) *KRAS* mRNA expression in rectal cancer tissue and adjacent normal mucosa. Box plot and *P*-value were produced using UALCAN. *P*=1.649440E-04.

**Figure 3:**
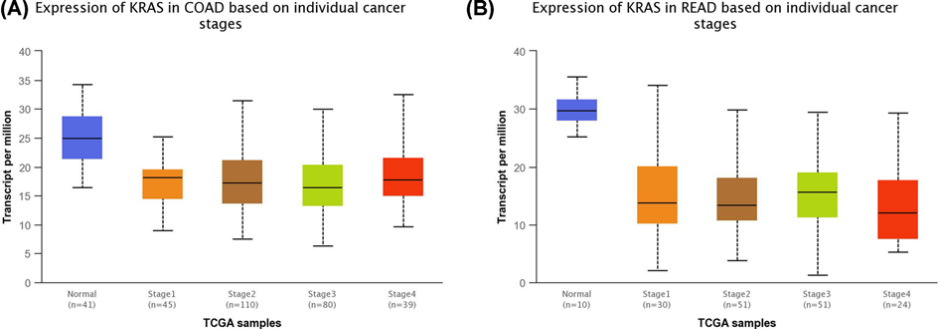
*KRAS* mRNA expression in samples for CRC based on individual cancer stages from TCGA dataset (**A**) *KRAS* expression in samples for colon cancer based on individual cancer stages. Using the UALCAN data, the expression of KRAS in colon cancer samples at any stages was lower than that in normal samples. Normal-vs-Stage1, *P*=6.19280005054179E-09; Normal-vs-Stage2, *P*=1.27364785384998E-12; Normal-vs-Stage3, *P*=1.37959976775903E-10; Normal-vs-Stage4, *P*=1.58269999999128E-05. (**B**) *KRAS* expression in samples for rectal cancer based on individual cancer stages. Using the UALCAN data, the expression of KRAS in rectal cancer samples at any stages was lower than that in normal samples. Normal-vs-Stage1, *P*=2.022800E-01; Normal-vs-Stage2, *P*=3.70320000000479E-05; Normal-vs-Stage3, *P*=1.815890E-04; Normal-vs-Stage4, *P*=1.881260E-03.

We performed a statistical comparison of *KRAS* expression from multiple CRC studies published in Oncomine database. Seven independent microarray studies comprising a total of 578 CRCs and 179 normal colorectal mucosa samples were evaluated from meta-analysis data by Oncomine. Meta-analysis identified that *KRAS* mRNA was under-expressed in CRC tissue (median rank = 2534.5, *P*=0.001). Based on the UALCAN data, *KRAS* mRNA expression was down-regulated in CRC tissue (Figure 2A,B). The expression of *KRAS* in CRC samples at any stages was lower than that in normal samples (Figure 3A,B).

### Polymorphisms of miR-143, miR-145, and *KRAS* 3′UTR and risk of CRC

*KRAS* rs712 and rs1137196, miR-143 rs41291957, and miR-145 rs74693964 and rs80026971 were genotyped in the present study. The genotype distribution of the five SNPs in the control group all conformed to Hardy–Weinberg equilibrium; their associations with the risk of CRC are presented in Table 2. As shown in the table, subjects with the heterozygous genotype CT of rs74693964 were more than twice as likely to have CRC as subjects with the wild genotype CC [adjusted odds ratio (*OR*) = 2.414, 95% *CI*: 1.385–4.206]. MiR-145 rs74693964 was associated with a significantly increased risk of CRC. However, *KRAS* rs712 and rs1137196, miR-143 rs41291957, and miR-145 rs80026971 showed no association with the risk of CRC. In the subgroup analysis, rs41291957 and rs74693964 were found to be associated with an increased risk of rectal cancer but not colon cancer (rs41291957 GA vs. AA: adjusted *OR* = 1.367, 95% *CI*: 1.005–1.860; rs74693964 CT vs. CC: adjusted *OR* = 2.820, 95% *CI*: 1.547–5.140) (Tables 2 and 3).

**Table 2:** Genetic association analyses of selected SNPs with CRC risk

Gene symbol	Genotype	Cases (*n*%)	Controls (*n*%)	*OR* (95% *CI*)^*^	*P-value*
KRAS	rs712				
	GG	301 (60.4%)	313 (64.1%)	1.0	
	GT	179 (35.9%)	159 (32.6%)	1.190 (0.911–1.555)	0.201
	TT	18 (3.6%)	16 (3.3%)	1.184 (0.591–2.372)	0.634
	G allele	781 (78.4%)	785 (80.4%)		
	T allele	215 (21.6%)	191 (19.6%)		
KRAS	rs1137196				
	CC	295 (59.7%)	299 (62.2%)	1.0	
	CA	180 (36.4%)	167 (34.7%)	1.108 (0.849–1.446)	0.452
	AA	19 (3.8%)	15 (3.1%)	1.303 (0.648–2.621)	0.458
	C allele	770 (77.9%)	765 (79.5%)		
	A allele	218 (22.1%)	197 (20.5%)		
miR-143	rs41291957				
	GG	208 (41.4%)	226 (46.1%)	1.0	
	GA	246 (48.9%)	210 (42.9%)	1.275 (0.979–1.662)	0.072
	AA	49 (9.7%)	54 (11%)	0.977 (0.634–1.505)	0.915
	G allele	662 (65.8%)	662 (67.5)		
	A allele	344 (34.2%)	318 (32.5)		
miR-145	rs74693964				
	CC	462 (91.1%)	470 (96.1%)	1.0	
	CT	45 (8.9%)	19 (3.9%)	2.414 (1.385–4.206)	0.002
	C allele	969 (95.6%)	959 (98.1%)		
	T allele	45 (4.4%)	19 (1.9%)		
miR-145	rs80026971				
	GG	497 (98%)	487 (98.6%)	1.0	
	GC	10 (2%)	7 (1.4%)	1.220 (0.457–3.258)	0.692
	C allele	1004 (99.0%)	981 (99.3%)		
	T allele	10 (1.0%)	7 (0.7%)		

* *OR* (95% *CI*) adjusted by age, sex, and family history of cancer.

**Table 3:** Genetic association analyses of selected SNPs with colon cancer and rectal cancer risk

Gene symbol	Genotype	Colon cancer (*n*=209)				Rectal cancer (*n*=298)			
		Cases (*nn*%)	Controls (*n*%)	*OR* (95% *CI*)^*^	*P-value*	Cases (*n*%)	Controls (*n*%)	*OR* (95% *CI*)^*^	*P-value*
KRAS	rs712								
	GG	118 (57.8)	315 (64.3)	1.0		183 (62.2)	315 (64.3)	1.0	
	GT	74 (36.3)	159 (32.4)	1.260 (0.889–1.787)	0.194	105 (35.7)	159 (32.4)	1.147 (0.842–1.562)	0.385
	TT	12 (5.9)	16 (3.3)	1.998 (0.916–4.362)	0.082	6 (2.0)	16 (3.3)	0.654 (0.250–1.712)	0.387
KRAS	rs1137196								
	CC	113 (55.9)	301 (62.3)	1.0		182 (62.3)	301 (62.3)	1.0	
	CA	78 (38.6)	167 (34.6)	1.269 (0.897–1.796)	0.179	102 (34.9)	167 (34.6)	1.010 (0.741–1.377)	0.949
	AA	11 (5.4)	15 (3.1)	1.943 (0.864–4.372)	0.108	8 (2.7)	15 (3.1)	0.913 (0.377–2.211)	0.840
miR-143	rs41291957								
	GG	88 (42.3)	226 (45.9)	1.0		120 (40.7)	226 (45.9)	1.0	
	GA	97 (46.6)	212 (43.1)	1.177 (0.833–1.663)	0.355	149 (50.5)	212 (43.1)	1.367 (1.005–1.860)	0.046
	AA	23 (11.1)	54 (11.0)	1.075 (0.621–1.862)	0.796	26 (8.8)	54 (11.0)	0.904 (0.537–1.522)	0.704
miR-145	rs74693964								
	CC	194 (92.8)	472 (96.1)	1.0		268 (89.9)	472 (96.1)	1.0	
	CT	15 (7.2)	19 (3.9)	1.901 (0.943–3.835)	0.073	30 (10.1)	19 (3.9)	2.820 (1.547–5.140)	0.001
miR-145	rs80026971								
	GG	207 (99.0)	489 (98.6)	1.0		290 (97.3)	489 (98.6)	1.0	0.318
	GC	2 (1.0)	7 (1.4)	0.608 (0.124–2.976)	0.540	8 (2.7)	7 (1.4)	1.694 (0.602–4.765)	

* *OR* (95%*CI*) adjusted by age, sex, and family history of cancer.

When stratified by smoking status, we found that the genotype distributions of miR-143 rs41291957 among non-smokers differed significantly between cases and controls. Compared with the GG genotype, those carrying heterozygous genotype GA had a nearly 40% increased risk for developing CRC (adjusted *OR* = 1.397, 95% *CI*: 1.007–1.936). In non-smokers, miR-145 rs74693964 remained a significant risk factor for CRC among subjects carrying the CT genotype (adjusted *OR* = 3.086, 95% *CI*: 1.468–6.484). Interaction analyses of the two SNPs and tobacco smoking were conducted using a multiplicative model; neither interaction effect showed statistical significance (Table 4).

**Table 4:** Association of selected SNPs with CRC risk after stratification by smoking status

Gene symbol	Genotype	Smoker				Non-smoker			
		Cases (*n*%)	Controls (*n*%)	*OR* (95% *CI*)^*^	*P-value*	Cases (*n*%)	Controls (*n*%)	*OR* (95% *CI*)^*^	*P-value*
KRAS	rs712								
	GG	108 (60.3%)	100 (66.7%)	1.0		193 (60.5%)	209 (63.0)	1.0	
	GT	66 (36.9%)	48 (32.0%)	1.361 (0.848–2.184)	0.202	113 (35.4%)	109 (32.8)	1.112 (0.799–1.546)	0.529
	TT	5 (2.8%)	2 (1.3%)	2.358 (0.444–12.514)	0.314	13 (4.1%)	14 (4.2)	0.960 (0.438–2.106)	0.920
	GT+TT	71 (39.7%)	50 (33.3%)	1.402 (0.880–2.232)	0.155	126 (39.5%)	123 (37.0)	1.094 (0.796–1.504)	0.579
Multiplicative interaction								1.263 (0.725–2.203)	0.410
KRAS	rs1137196								
	CC	105 (59.7)	98 (66.7)	1.0		190 (59.7)	198 (60.2)	1.0	
	CA	65 (36.9)	47 (32.0)	1.354 (0.841–2.180)	0.212	115 (36.2)	118 (35.9)	1.003 (0.723–1.391)	0.986
	AA	6 (3.4)	2 (1.4)	2.835 (0.555–14.479)	0.210	13 (4.1)	13 (4.0)	1.001 (0.449–2.229)	0.999
	CA+AA	71 (40.3)	49 (33.4)	1.415 (0.887–2.259)	0.145	128 (40.3)	131 (39.9)	1.003 (0.730–1.377)	0.987
Multiplicative interaction								1.423 (0.814–2.487)	0.215
miR-143	rs41291957								
	GG	78 (43.1%)	66 (44.0%)	1.0		130 (40.4%)	157 (46.9%)	1.0	
	GA	83 (45.9%)	67 (44.7%)	1.079 (0.677–1.721)	0.748	163 (50.6%)	141 (42.1%)	1.397 (1.007–1.936)	0.045
	AA	20 (11.0%)	17 (11.3%)	0.956 (0.460–1.986)	0.905	29 (9.0%)	37 (11.0%)	0.918 (0.533–1.580)	0.756
	GA+AA			1.053 (0.677–1.640)	0.818			1.296 (0.949–1.770)	0.103
Multiplicative interaction								0.805 (0.470–1.378)	0.429
miR-143	rs74693964								
	CC	167 (91.3%)	140 (94.0%)	1.0		295 (91.0%)	324 (97.0%)	1.0	
	CT	16 (8.7%)	9 (6.0%)	1.672 (0.687–4.070)	0.257	29 (9.0%)	10 (3.0%)	3.086 (1.468–6.484)	0.003
Multiplicative interaction							0.473 (0.153–1.462)	0.194	
miR-145	rs80026971								
	GG	181 (98.9%)	150 (99.3)	1.0		316 (97.5%)	331 (98.2%)	1.0	
	GC	2 (1.1%)	1 (0.7)	1.290 (0.113–14.738)	0.838	8 (2.5%)	6 (1.8%)	1.199 (0.406–3.539)	0.742
Multiplicative interaction								1.141 (0.080–16.168)	0.922

* *OR* (95% *CI*) adjusted by age, sex, and family history of cancer.

Although miR-143 and miR-145 are located close to each other on 5q33, our analysis showed no linkage disequilibrium between them. To evaluate the potential cumulative effects of miR-143 and miR-145, we defined at-risk genotypes as those with *OR* values greater than 1 under a dominant model of rs41291957 and rs74693964. We compared the distributions of the number of at-risk genotypes between cancer cases and controls. The risk of having CRC increased with the number of at-risk genotypes (*P***_trend_**=0.003). When split by cancer location, individuals harboring two at-risk genotypes had an increased risk of rectal cancer relative to those with none (*OR* = 3.738, 95% *CI*: 1.725–8.101) (Table 5).

**Table 5:** Genetic association analyses of number of at-risk genotypes within rs41291957 and rs74693964 with colon cancer and rectal cancer risk

Number of at-risk genotypes	Colon cancer				Rectal cancer				CRC			
	Cases (*n*%)	Controls (*n*%)	*OR* (95% *CI*)^*^	*P-value*	Cases (*n*%)	Controls (*n*%)	*OR* (95% *CI*)^*^	*P-value*	Cases (*n*%)	Controls (*n*%)	*OR* (95% *CI*)^*^	*P-value*
0	106 (51.0)	270 (55.4)	1.0		136 (46.1)	270 (55.4)	1.0		242 (48.1)	270 (55.4)	1.0	
1	92 (44.2)	206 (42.3)	1.151 (0.824–1.607)	0.411	139 (47.1)	206 (42.3)	1.378 (1.026–1.874)	0.033	231 (45.9)	206 (42.3)	1.273 (0.984–1.647)	0.066
2	10 (4.8)	11 (2.3)	2.242 (0.918–5.475)	0.076	20 (6.8)	11 (2.3)	3.738 (1.725–8.101)	0.001	30 (6.0)	11 (2.3)	3.032 (1.479–6.215)	0.002
*P*_trend_				0.128				0.001				0.003

* *OR* (95%*CI*) adjusted by age, sex, and family history of cancer.

### Polymorphisms of miR-143, miR-145, and *KRAS* 3′UTR and CRC survival

A totsl of 222 CRC cases with Hangzhou household registration had survival data extracted from the Cancer Registration System and Death Surveillance System of Hangzhou Center for Disease Control and Prevention. Of the 222 patients with CRC recruited between May 2014 and May 2015, 34 died of CRC by 1 January 2019. Associations between polymorphisms of miR-143, miR-145, and the *KRAS* 3′UTR and survival of patients with CRC were explored. First, we used the Kaplan–Meier method to compare OS among different genotypes of selected SNPs. Then, adjusted hazard regressions (HRs) were obtained by Cox regression analysis for further confirmation of the relationships between the genotypes and survival of patients with CRC. The results showed that the mutant homozygote TT of rs712 was associated with decreased survival time of patients with CRC (log-rank test, *P*=0.044). Compared with the reference genotype GG of rs712, the CRC cases with TT genotype had a significant increase in number of deaths (adjusted *HR* = 4.328, 95% *CI*: 1.236–15.147). The polymorphisms of miR-143 and miR-145 did not show any statistical association with survival of patients with CRC (Table 6).

**Table 6:** Kaplan–Meier survival estimation of mean survival and HRs of selected SNPs

Gene symbol	Genotypes	*n*%	Mean survival (in months)	Log rank, *P-value*	*HR* (95% *CI*)^*^, *P-value*
KRAS	rs712				
	GG	137 (62.5)	51.94	0.044	1.0
	GT	74 (33.8)	48.66		1.672 (0.815–3.432), 0.161
	TT	8 (3.7)	37.00		4.328 (1.236–15.147), 0.022
KRAS	rs1137196				
	CC	131 (61.5)	52.28	0.098	1.0
	CA	75 (35.2)	47.49		1.860 (0.913–3.788),0.088
	AA	7 (3.3)	41.29		3.030 (0.676–13.578),0.147
miR-143	rs41291957				
	GG	93 (42.5)	47.49	0.123	1.0
	GA	105 (47.9)	52.07		0.573 (0.280–1.174),0.128
	AA	21 (9.6)	48.82		0.375 (0.078–1.613),0.188
miR-145	rs74693964				
	CC	201 (90.5)	51.14	0.441	1.0
	CT	21 (9.5)	49.43		0.491 (0.117–2.056),0.330
miR-145	rs80026971				
	GG	218 (98.2)	51.34	0.557	1.0
	GC	4 (1.8)	41.25		1.481 (0.195–11.235),0.704

* *HR* (95% *CI*) adjusted by age, sex, and tumor stage.

## Discussion

MiR-143 and miR-145 which are located on 5q23 which may originate from the same primary miRNA. Michael et al. showed that miR-143 and miR-145 displayed consistently decreased expression levels of mature miRNA at the colorectal neoplasm when compared with healthy colorectal mucosa. Several other studies have confirmed this finding [9,21]. The present study found that both miR-143/145 showed reduced expression in rectal cancer when compared with adjacent non-neoplastic large intestinal mucosa based on microarray gene expression datasets from GEO, which is consistent with previous studies. *KRAS* is one of the most frequently mutated genes in CRC risk. A number of recent studies have demonstrated the significance of *KRAS* mutation in CRC carcinogenesis [15,22]; however, *KRAS* gene expression status in CRC has been less reported. In view of this, we conducted a meta-analysis for *KRAS* gene expression from multiple CRC studies published in Oncomine database. We found that *KRAS* expression was down-regulated in CRC tissues in four stages of the cancer. Mazza et al. evaluated of the miRNAome and transcriptome of matched pairs of tumor and adjacent non–neoplastic large intestinal mucosa samples of CRC. They noted concurrent down-regulation of *KRAS* and the miR-143/145 cluster in CRC tissue [16]. This result was interpreted in terms of a feed-forward mechanism in which the miR-143/145 polycistronic cluster targets the RAS-responsive element-binding protein RREB1 and *KRAS*, which, in turn, induce down-regulation of the cluster [14].

Emerging evidence has shown that miRNA-related SNPs may alter an individual’s susceptibility to CRC by disrupting miRNAs’ process, expression, or interaction with target mRNA [23]. However, no SNP of the miR-143 and miR-145 genes could be identified by the HapMap and SNP database (dbSNP) retrieval. Thus we selected the SNPs within the miRNA regulatory region/transcription factor-binding sites for further study. MiR-145 rs74693964 is located 65 bp downstream of miR-145. According to functional predictions based on HaploReg annotations [24] and the RegulomeDB database [25], this SNP has been identified as a promoter histone modification or enhancer histone modification region in more than 20 tissues, including colonic mucosa and rectal mucosa. In the present study, individuals with the CT genotype of rs74693964 in the Chinese population had a two-fold increased risk for having CRC when compared with those carrying the CC genotype. After stratification by smoking status, miR-145 rs74693964 was found to be significantly associated with an increased risk of CRC among patients who were non-smokers. To date, only two studies have reported an association of miR-145 rs74693964 with risk of cancer; one was a study of cervical cancer and the other of non-small-cell lung cancer [26,27]. No similar study involving CRC has yet been reported. To our knowledge, the present work is the first investigation of the link between miR-145 rs74693964 and risk of CRC in the Chinese population.

In previous studies, Li et al. [28] reported a significant effect of mutant genotypes or alleles of rs41291957 on risk of having CRC, On the other hand, Ying et al. [29] failed to find any association between rs41291957 and susceptibility to CRC. In our study, risk of rectal cancer was shown to be associated with the rs41291957 heterozygous genotype. Rs41291957 is located 91 base pairs (bp) upstream of miR-143. Saini et al. demonstrated that up to 60% of miRNAs have transcription factor binding sites (TFBSs) within 1 kilobases (kb) of the start of the pre-miRNA [30], indicating that rs41291957 in the promoter region may be involved in the transcriptional activation of miR-143. Furthermore, bioinformatic predictions using HaploReg and RegulomeDB indicated that rs41291957 is probably involved in epigenetic modifications that promote colorectal tumorigenesis.

In the present study, the cumulative effects of significant polymorphisms of miR-143 and miR-145 were evaluated in CRC. The risk of CRC increased with the number of at-risk genotypes, especially in rectal cancer. The average SNP density of clustered miRNAs was significantly lower than that of the individual miRNAs, which may to some degree reflect the critical biological functions regulated by clustered miRNAs [31]. The miR-143/145 cluster co-ordinately plays an important part in the carcinogenesis of CRC [32]. It is thus a reasonable assumption that the more mutations occur in the miR-143/145 cluster, the greater the risk of CRC.

*KRAS* is a direct target of miR-143/145. In the present study, no SNP was identified within the binding region of miR-143/145, nor was there any association with risk of having CRC. However, our results indicated that the rs712 G>T polymorphism in the 3′UTR of the *KRAS* gene may modulate survival outcome of patients with CRC. Multiple miRNAs, including miR-200b, miR-200c, and miR-429, target rs712. The miR-200 family (miR-200b, miR-200c, and miR-429) has been widely investigated with regard to its role in tumor metastasis and regulating cancer stem cells [33]. Pichler et al. found that miR-200 family expression was associated with poor prognosis in patients with CRC and with cancer stem cell properties in CRC [34]. Therefore, the rs712 G>T change might attenuate its binding capacity with the miR-200 family. Although the association between the *KRAS* rs712 polymorphism and cancer risk has been widely studied [35–37], the effects of this polymorphism on survival of patients with CRC are still unclear. Schneiderova et al. [38] indicated that individuals with colon cancer carrying the heterozygous GT genotype had longer OS. On the other hand, the survival impact of rs712 on survival of patients with CRCl was not significant in a study by Dai and colleagues[39]. Our study suggests that a poor prognosis in Chinese patients with CRC is associated with the homozygous TT genotype. The limited and conflicting results on the prognostic value of *KRAS* rs712 as a predictor for survival of patients with CRC indicate that more studies in different populations are required.

There were some limitations to the present study. First, owing to the lack of RNA samples for the study population, we were unable to carry out functional validation tests. The biological functions of the selected SNPs in CRC were inferred and predicted using the available online tools. Second, the participants in the cancer group and control group were collected from a hospital and from the community, respectively; thus, selection bias cannot be ignored. Finally, the relatively small sample size, especially for the survival analysis, may have hindered the ability of the study to detect weak gene-disease associations and gene–environment interactions.

## Conclusions

In conclusion, our results suggest that rs74693964 C/T and rs41291957 G/A in the miR-143/145 cluster might have cumulative effects on risk of rectal cancer. Rs712 G/T in *KRAS* might be associated with poorer survival in CRC. Further large population-based prospective studies as well as functional validation are warranted to advance our understanding of the role of these factors in CRC.

## Supplementary Material

Supplementary Table S1

## Data Availability

The analysed datasets generated during the study are available from the corresponding author on reasonable request.

## Abbreviations

CRC: colorectal cancer
GEO: Gene Expression Omnibus
HR: hazard ratio
miRNA: microRNA
ncRNA: non-coding RNA
OR: odds ratio
OS: overall survival
SNP: single nucleotide polymorphism
TagSNP: tag single nucleotide polymorphism
3′UTR: 3′ Untranslated Region
