# Supplementary material for: Association analysis of miRNA-related genetic polymorphisms in miR-143/145 and KRAS with colorectal cancer susceptibility and survival
Source: Biosci Rep. 2021 Apr 22;41(4):BSR20204136. doi: 10.1042/BSR20204136 (PMC8062955; doi:10.1042/BSR20204136)
Supplement: Supplementary Table S1 [file BSR-2020-4136_supp.pdf]

Supplementary table Descriptions of selected SNPs of miR-143, miR-145 and Kras

SNP	Gene	SNP Location	Allelic change	Region
rs41291957	mir-143	5:148808390	G/A	-91bp upstream of pre-mir-143
rs191479100*	mir-143	5:148808678	G/T	92bp downstream of pre-mir-143
rs80026971	mir-145	5:148810010	G/C	-199bp upstream of pre-mir-145
rs74693964	mir-145	5:148810361	C/T	65bp downstream of pre-mir-145
rs712	*KRAS*	12:25209618	G/T	3'UTR of *KRAS*
rs1137196	*KRAS*	12:25206035	C/A	3'UTR of *KRAS*

* Of the 1012 DNA samples, only 3 samples were GT Heterozygous, the others were all GG homozygous, data were not shown in the article.
